# Identification of PTK2 as an adverse prognostic biomarker in breast cancer by integrated bioinformatics and experimental analyses

**DOI:** 10.3389/fmolb.2022.984564

**Published:** 2022-12-01

**Authors:** Yanru Chen, Wei Wang, Lingyu Fang, Zhenyang Zhang, Shishan Deng

**Affiliations:** ^1^ North Sichuan Medical College, Institute of Basic Medicine and Forensic Medicine, Sichuan, China; ^2^ Sichuan Key Laboratory of Medical Imaging and school of Medicine Imaging, North Sichuan Medical College, Sichuan, China; ^3^ Department of Academician (expert) Workstation, Biological Targeting Laboratory of Breast Cancer, Breast and Thyroid Surgery, Affiliated Hospital of North Sichuan Medical College, Sichuan, China; ^4^ Wuxi School of Medicine, Jiangnan University, Jiangsu, China

**Keywords:** breast cancer, PTK2, prognosis, immune infiltrates, biomarker

## Abstract

PTK2 is highly expressed in many cancers and is involved in cell growth, survival, migration, and invasion. However, the prognostic value of PTK2 and its potential function remain unclear in breast cancer. Therefore, we performed a comprehensive analysis of multiple public databases to explore the roles of PTK2. By integrating multiple datasets, we found that PTK2 mRNA expression in breast cancer tissue was higher than that in normal breast tissue or adjacent tissue. High PTK2 expression was associated with lymph node metastasis stage, tumor stage, breast cancer type, age, TP53 mutation, and gender and significantly predicted a poor survival outcome in breast cancer patients. Gene Ontology (GO) and Kyoto Encyclopedia of Genes and Genomes (KEGG) results suggested that PTK2 and co-expressed genes participated in the cell cycle. Immune infiltration analysis clarified that high PTK2 expression was positively correlated with infiltrating levels of CD8^+^ T cells, CD4^+^ T cells, macrophages, neutrophils, and dendritic cells. The DNA methylation of PTK2 in breast cancer tissues was higher than that in normal tissues, and high PTK2 methylation was correlated with poor prognosis in breast cancer patients. Furthermore, 16 possible ceRNA networks related to PTK2 were constructed for breast cancer. Additionally, PTK2 knockdown could suppress the proliferation and migration ability of MCF-7 cells. These results suggest that PTK2 can be used as a prognostic biomarker for breast cancer.

## Introduction

Breast cancer is the most common cancer that threatens women’s health worldwide (Anastasiadi et al., 2017). Numerous studies have made tremendous strides in the breast cancer field; breast cancer patients have been able to receive treatment with a combination of surgery, chemotherapy, and radiation therapy. Although personalized treatment protocols have been used to improve overall survival, breast cancer remains a major public health problem, and its morbidity and mortality rates are expected to increase significantly in the coming years (Sieg et al., 2000; Batista et al., 2014). Thus, the search for new diagnostic and prognostic biomarkers of breast cancer remains of great importance.

Nonreceptor protein tyrosine kinase 2 (PTK2), also known as focal adhesion kinase (FAK), regulates the signal transduction of integrin and growth factor receptors (Sieg et al., 2000; Zhao and Guan, 2011; Batista et al., 2014). Activated PTK2 can regulate multiple cellular functions, including cell adhesion, proliferation, and migration (Miyasaka et al., 2001; Itoh et al., 2004; McLean et al., 2005; Zhao and Guan, 2011; Batista et al., 2014; Sulzmaier et al., 2014). Previous studies have shown that PTK2 overexpression may be associated with tumor cell migration and activation of the extracellular-signal-regulated kinase signaling pathway. A previous study showed that PTK2 activates the survival signaling pathway in breast cancer and may inhibit tumor growth (Sethuraman et al., 2016). In addition, PTK2 is overexpressed in many major cancer types, including lung cancer, hepatocellular carcinoma, and lymphocytic leukemia (Weisser et al., 2014; Fan et al., 2019). Upregulation of PTK2 expression in cancer has been linked to malignant progression and poor prognosis (Miyasaka et al., 2001; Fujii et al., 2004; Itoh et al., 2004; McLean et al., 2005). Moreover, the expression of PTK2 genes in hepatocellular carcinoma is inversely correlated with its promoter methylation level. As a prototypic oncogene, PTK2 can regulate cancer stem cells to enhance the tumorigenesis of hepatocellular carcinoma (Fan et al., 2019). However, the exact function and mechanism of PTK2 in breast cancer remain unknown.

In this study, we used various public databases to explore the differential expression of PTK2 in breast cancer tissues and normal tissues, its diagnostic value in breast cancer, and its correlation with clinicopathological parameters. Multidimensional analysis was used to evaluate the gene alterations and gene and protein functional networks related to the expression of PTK2 in breast cancer and to explore the relationship between its differential expression and methylation and the ceRNA regulatory network. Our study provides novel insights that show the potential role of PTK2 in breast cancer and its potential role as a prognostic biomarker.

## Materials and methods

### PTK2 expression across human cancers in TIMER

TIMER (https://cistrome.shinyapps.io/timer/) is a website that enables comprehensive analysis of gene expression in various cancers (Li et al., 2017). The TIMER website allows users to explore gene expression profiles in tumor and normal tissues. In this database, 10,897 samples across 32 cancer types from the TCGA dataset were integrated to estimate the abundance of immune infiltrates. We used the “Diff Exp module” to analyze PTK2 expression in various types of cancer. The Wilcoxon test was used to assess differential PTK2 expression. In addition, the “Correlation module” was used to assess the correlations between PTK2 transcription levels and immune cell infiltration, including B cells, neutrophils, CD4^+^ T cells, macrophages, CD8^+^ T cells, and dendritic cells. The correlation between PTK2 expression and immune infiltration was evaluated using Spearman’s correlation.

### Correlations between PTK2 expression and clinicopathological parameters in UALCAN

The UALCAN online database (http://ualcan.path.uab.edu/index.html) is a website for effective online analysis and mining of cancer data. The database enables analysis of TCGA RNA-sequencing results (TPM data) of 31 tumors and differential expression of 31 malignant and normal tissues. In addition, biomarker identification, expression profiling, survival analysis, etc., can also be performed (Chandrashekar et al., 2017). In this study, we analyzed PTK2 expression differences between breast cancer tissue and normal tissue, the correlation between PTK2 mRNA expression and clinicopathological parameters, and the methylation levels of PTK2 in a breast cancer dataset. Wilcoxon tests were performed to analyze the association between clinicopathological parameters and PTK2 mRNA expression in breast cancer.

### TCGA data acquisition

The Cancer Genome Atlas Project (TCGA) is an effort by the National Cancer Institute. The TCGA database includes whole-genome sequences (WGS), survival data, methylation, RNA expression, proteomics, and clinical data (Chandran et al., 2016). The RNA sequence data and the corresponding clinicopathological information for 1,109 breast cancer patients were downloaded from the TCGA database (https://portal.gdc.cancer.gov/). The fragments per kilobase per million (FPKM) data were transformed to TPM (transcriptions per million reads) for the analyses. In this study, we analyzed the mRNA expression of PTK2 in breast cancer and normal tissues from the TGCA breast cancer database.

### Human Protein Atlas

The Human Protein Atlas (HPA) (https://www.proteinatlas.org/) is a public database that uses transcriptomic and proteomic technologies to study protein expression in different human tissues and organs at the RNA and protein levels (Thul and Lindskog, 2018). In this study, we used the “Tissue Atlas” and “Cell Line Atlas” to show the distribution of PTK2 in human tissues and cell lines.

### PrognoScan database analysis

The PrognoScan database (http://www.abren.net/PrognoScan/) explores the gene expression and clinical prognosis of patients by collecting a large number of publicly available cancer microarray datasets (Mizuno et al., 2009). We used the PrognoScan database to analyze the prognostic value of PTK2 expression in breast cancer patients using parameters such as overall survival (OS) and relapse-free survival (RFS). The threshold was adjusted to a Cox *p*-value < 0.05.

### Gene alteration in cBioPortal

The cBio Cancer Genomics Portal (cBioPortal, www.cbioportal.org) is an open-access resource that explores multidimensional cancer genomics datasets from more than 5,000 tumor samples from 20 cancer studies (Fang et al., 2020). We selected log RNA Seq V2 RSEM data from TCGA-breast cancer on this website to perform a mutation analysis and co-expression gene analysis. The genomic alteration types and alteration frequency of PTK2 in breast cancer were analyzed through the “OncoPrint module”. The Kaplan‒Meier survival curve of PTK2 was analyzed through the “Comparison/Survival” module in cBioPortal.

### GeneMANIA and STRING analysis

The STRING database can provide researchers with the mechanism of disease development and can also explore protein–protein interactions (von Mering et al., 2003). In addition, GeneMANIA is capable of constructing complex gene–gene function interaction networks from gene lists (Montojo et al., 2014). Therefore, we used the GeneMANIA database (http://www.genemania.org) and STRING online database (https://string-db.org/) to construct the gene–gene interaction network and protein–protein interaction network, respectively.

### LinkedOmics analysis

The LinkedOmics online database (http://www.linkedomics.org/login.php) is a multi-omics database that integrates mass spectrometry (MS) proteomic data for selected TCGA tumor samples (Vasaikar et al., 2018). Moreover, the LinkedOmics online tool contains multi-omics and clinical data for 32 malignancies and more than 10,000 patients. The Pearson correlation coefficient was used for statistical analysis of PTK2 co-expression and is represented by a volcano map. The PTK2 co-expression genes are represented by a heatmap. Gene Ontology analysis and KEGG pathway analysis of PTK2 and its co-expression genes were accomplished using the gene set enrichment analysis (GSEA) module. The rank criterion was a false discovery rate (FDR) < 0.05.

### MethSurv analysis

MethSurv (https://biit.cs.ut.ee/methsurv/) is a web tool for survival analysis based on CpG methylation patterns using TCGA data for 25 different types of cancer and 7,358 patients (Modhukur et al., 2018). The DNA methylation of PTK2 at CpG sites and the prognostic value of these CpG sites in breast cancer were analyzed using MethSurv.

### Prediction of lncRNAs and ceRNA network construction

The target miRNAs of PTK2 were predicted using the starBase 3.0 database (www.starbase.sysu.edu.cn), and the prediction results included analysis from the following databases: PITA, miRmap, and TargetScan. In addition, the TargetScan (http://www.targetscan.org/vert_72) database was used to predict potential binding sites for target miRNAs and PTK2. Finally, we used the miRNet2.0 (www.mirnet.ca/miRNet/home.xhtml) database and starBase online analysis tool to predict the target lncRNAs of miRNAs. We also established the key lncRNA–miRNA–mRNA (PTK2) ceRNA network for breast cancer.

### Cell culture and transfection

Human breast cancer cell lines (MCF-7, BT-549, and MDA-MB-231) and normal human mammary epithelial cell line (MCF-10A) were obtained from Precell Biotechnology Co., Ltd. All cell lines were cultured in DMEM with 10% fetal bovine serum (FBS; C0400, VivaCell, Shanghai, China) and 1% penicillin and streptomycin (C0222, Beyotime, China) and incubated at 37°C and 5% CO_2_ in a humidified incubator. The MCF-7 cells were seeded in a 6-well plate at 2,000 cells per well and incubated for 16 h. The MCF-7 cells were then transfected with the specific small interfering RNAs (siRNAs) using JetPrime^®^ transfection reagent (114–15, Polyplus Transfection, United States) according to the protocol of the manufacturer. The siRNA sequences were as follows: Human si-PTK2 #1: 5′- GCC​CAG​GUU​UAC​UGA​ACU​UAA -3′; Human si-PTK2 #1: 5′- GAU​GUU​GGU​UUA​AAG​CGA​UUU -3′.

### Western blot

The cell protein was homogenized in RIPA buffer, and then, the protein was quantified by bicinchoninic acid assay (P0010, Beyotime, China). Total protein was separated using 8% SDS-PAGE gel and transferred to polyvinylidene difluoride membranes. The membranes were blocked with 5% skimmed milk in TBST for 2 h at room temperature. The membranes were incubated with the following primary antibodies at 4°C overnight: anti-FAK (1:1,000, ab40794, Abcam, United States) and anti-GAPDH (1:1,000, ab9485, Abcam, United States). After washing, the membranes were incubated with secondary antibodies for 2 h at room temperature and visualized by the enhanced chemiluminescence reagent (BL523, Biosharp, China). Relative protein expression levels were assessed using ImageJ software.

### RNA extraction and RT–qPCR assay

The total RNA was extracted using TRIzol reagent (15596018, Invitrogen, United States) according to the manufacturer’s protocol. Total RNA (1 μg) was retro-transcribed into cDNA using the HiScript III RT SuperMix for qPCR (+gDNA wiper) (R323-01, Vazyme, China). RT–qPCR was performed in LightCycler 96 (Roche, United States) according to instructions from TB Green^®^ Premix Ex Taq™ II (RR820A, Takara, Japan). GAPDH was used as a reference control. Relative quantification was performed using the 2^−ΔΔCT^ method. The primer sequences were as follows: PTK2 forward ACA​CAT​CTT​GCT​GAC​TTC​ACT​C, PTK2 reverse GAC​TGC​GAG​GTT​CCA​TTC​AC, GAPDH forward TGA​CTT​CAA​CAG​CGA​CAC​CCA, and GAPDH reverse CAC​CCT​GTT​GCT​GTA​GCC​AAA.

### Cell proliferation test

Cell proliferation was detected by using the Cell Counting Kit-8 (CCK8) (C0037, Beyotime, China). Briefly, the MCF-7 cells were seeded into a 96-well culture plate at 2,000 cells per well. The 450 nm absorbances of cells at 24, 48, and 72 h were examined according to the manufacturer’s instructions.

### Colony formation experiment

The MCF-7 cells were seeded into a 6-well culture plate at 5,000 cells per well and cultured for 5–10 days. Then, the medium was discarded, and cells were fixed with 4% paraformaldehyde for 20 min at room temperature. The cells were washed twice with phosphate buffer solution (PBS) before staining with crystal violet for 15 min at room temperature. The image was captured using a microscope. ImageJ software was used for analysis.

### Wound-healing assay

The MCF-7 cells were seeded into a 6-well culture plate at 30,000 cells per well. After the cells reached 100% confluence, the 200-μL yellow straw tip was used to make a straight line. Images were captured at 0, 12, and 24 h after making the scratch by using a microscope. The cell migration ability was assessed by calculating the scratch healing rate (the area of scratch at 0 h–the area of scratch at 24 h)/the area of scratch at 0 h.

### Statistical analysis

Statistical analysis was performed using the Statistical Package for Social Sciences (SPSS 19.0 for Windows, SPSS, Chicago, IL). The Wilcoxon test was used to analyze the relationships between clinicopathological characteristics and PTK2 expression. The survival curve results of PrognoScan and TCGA are displayed with HR and P or Cox *p* values from a log-rank test. The correlation between PTK2 expression and other genes or immune infiltration levels was evaluated by a Pearson correlation or Spearman correlation. Data were expressed as mean ± SEM of three independent repeats. One-way ANOVA with Tukey’s post hoc test was applied for multiple comparisons. *p* < 0.05 indicated statistically significant differences.

## Results

### PTK2 expression is increased in breast cancer patients

**FIGURE 1:**
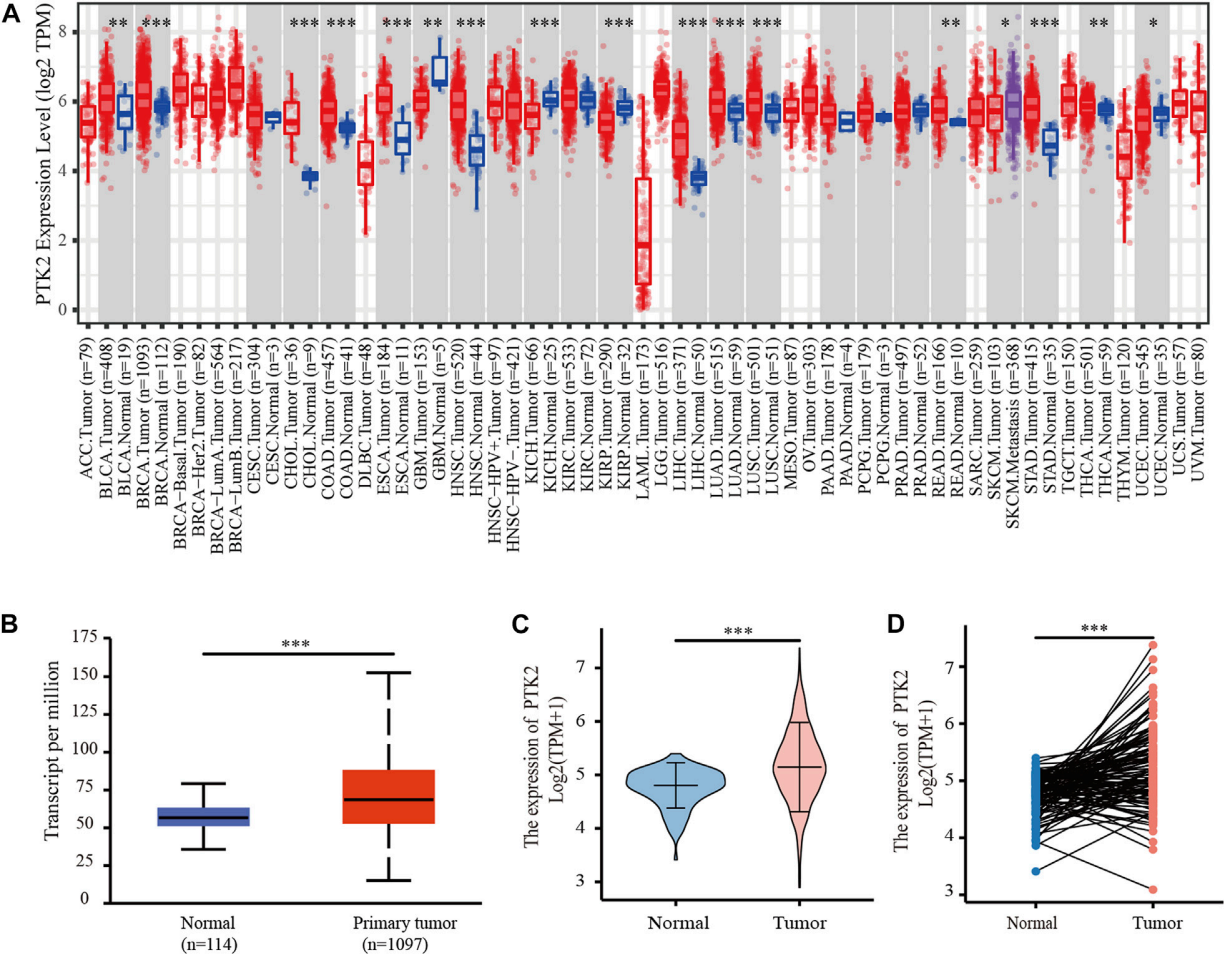
Expression of PTK2 in breast cancer. **(A)** Expression of PTK2 in different cancers in the TIMER database. **(B)** PTK2 expression in breast cancer was assessed by using the UALCAN database. **(C)** PTK2 expression levels in breast cancer from the TCGA database. **(D)** PTK2 expression in paired tissues. *p < 0.05 and ***p < 0.001.

PTK2 gene expression in human cancers and normal tissues was assessed using the TIMER online database. High PTK2 mRNA expression was observed in urothelial bladder carcinoma (BLCA), invasive breast carcinoma (BRCA), urothelial bladder carcinoma (BLCA), cholangiocarcinoma (CHOL), colon adenocarcinoma (COAD), esophageal carcinoma (ESCA), head–neck squamous cell carcinoma (HNSC), hepatocellular carcinoma (LIHC), lung adenocarcinoma (LUAD), rectum adenocarcinoma (READ), stomach adenocarcinoma (STAD), thyroid carcinoma (THCA), and uterine corpus endometrial carcinoma (UCEC) (Figure 1A). To further evaluate PTK2 expression in breast cancer, we used the UALCAN database for analysis. We found that PTK2 expression was higher in breast cancer tissues than in normal tissues (Figure 1B). In addition, we examined PTK2 mRNA expression in breast cancer using RNA-seq data from TCGA. PTK2 mRNA expression was significantly elevated in breast cancer tissues compared with that in adjacent normal tissues (Figure 1C). Furthermore, a significant increase in PTK2 expression was observed in paired tumor samples compared with adjacent normal samples (Figure 1D). PTK2 mRNA expression was relatively high in breast cancer cell lines (Supplementary Figure S1A). Analysis of the HPA database showed that PTK2 expression in normal breast tissue was higher than that in the majority of human tissues (Supplementary Figure S1B). In addition, the expression of PTK2 was highest in C-14 smooth muscle cells (Supplementary Figure S1C).

### PTK2 expression is associated with clinicopathological features in breast cancer

**FIGURE 2:**
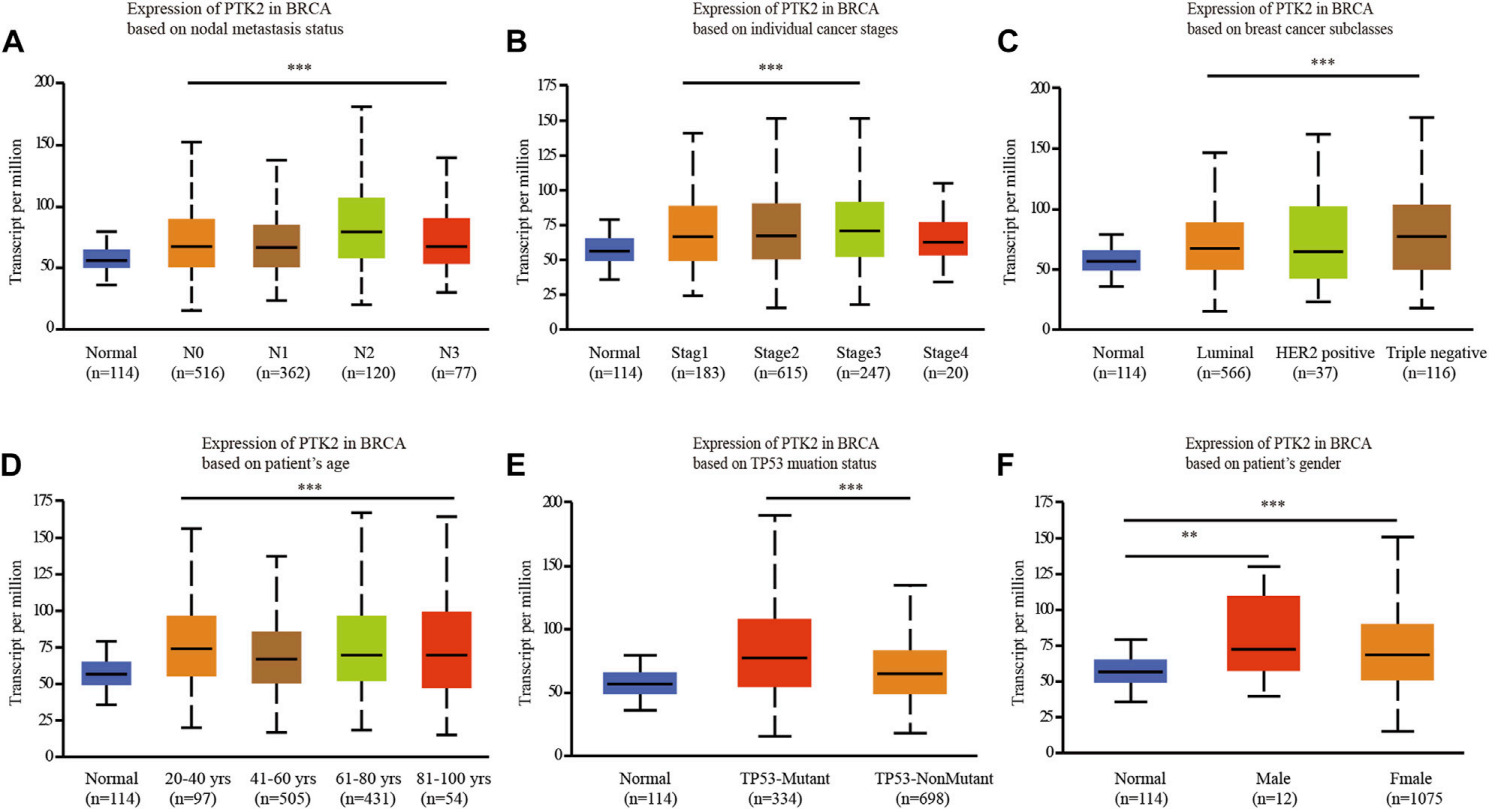
Box plot evaluating PTK2 expression of patients with breast cancer according to different clinical characteristics using the UALCAN database. **(A)** Nodal metastasis, **(B)** tumor stage, **(C)** breast cancer subclasses, **(D)** age, **(E)** T53 mutation status, and **(F)** gender.

The UALCAN online tool was used to analyze the association of PTK2 mRNA expression with clinicopathological parameters, including nodal metastasis status, breast cancer subclasses, age, sex, race, and menopausal status. PTK2 mRNA expression was significantly elevated in breast cancer patients compared with that in healthy individuals. Regarding cancer stages, a significant increase in PTK2 mRNA expression was observed in breast cancer patients in stages 1, 2, and 3. In terms of tumor histology, PTK2 mRNA expression was higher in patients with breast cancer classified as LDC, ILC, mixed, other, and medullary than the expression in other classification categories. The expression of PTK2 in LDCs was higher than those in ILCs, mucinous cells, and metaplastic cells. In addition, PTK2 mRNA expression was significantly increased in both TP53-mutant and TP53-nonmutant breast cancer patients compared with that in normal controls. The expression of PTK2 in TP53-mutant cells was higher than that in TP53-nonmutant cells (Figure 2).

### High PTK2 expression is associated with poor prognosis in breast cancer

**FIGURE 3:**
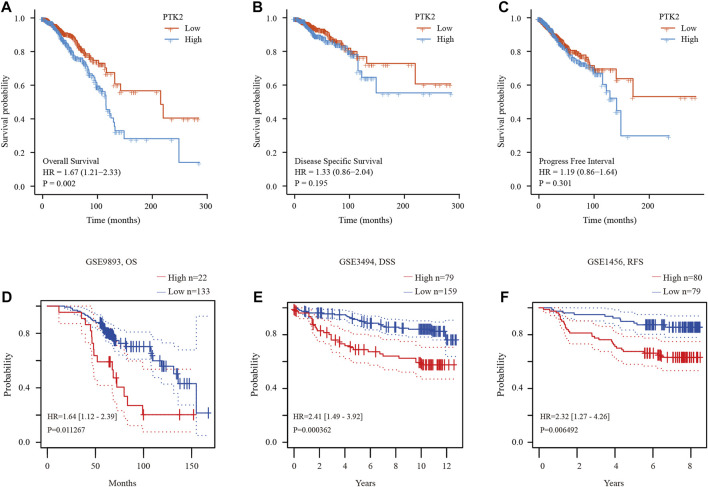
Prognostic value of PTK2 in breast cancer. **(A–C)** Survival curves of OS, DSS, and PFI from the TCGA database. **(D–F)** Survival curves using the PrognoScan database are shown for OS, DSS, and RFS.

The expression level of PTK2 is closely related to breast cancer progression. We subsequently tested the prognostic value of PTK2. According to the Kaplan‒Meier survival curves, breast cancer patients with high PTK2 expression exhibited poor overall survival (OS) (Figure 3A), but disease-specific survival (DSS) and progression-free interval (PFI) were not affected (Figures 3B,C). Furthermore, according to the PrognoScan online tool, elevated PTK2 expression was significantly associated with poorer OS, DSS, and recurrence-free survival (RFS) in the GSE9893, GSE3494, and GSE1456 cohorts, respectively (Figures 3D–F). The results suggest that PTK2 is significantly correlated with the prognosis of breast cancer.

### Genomic alterations of PTK2 and the gene and protein networks

**FIGURE 4:**
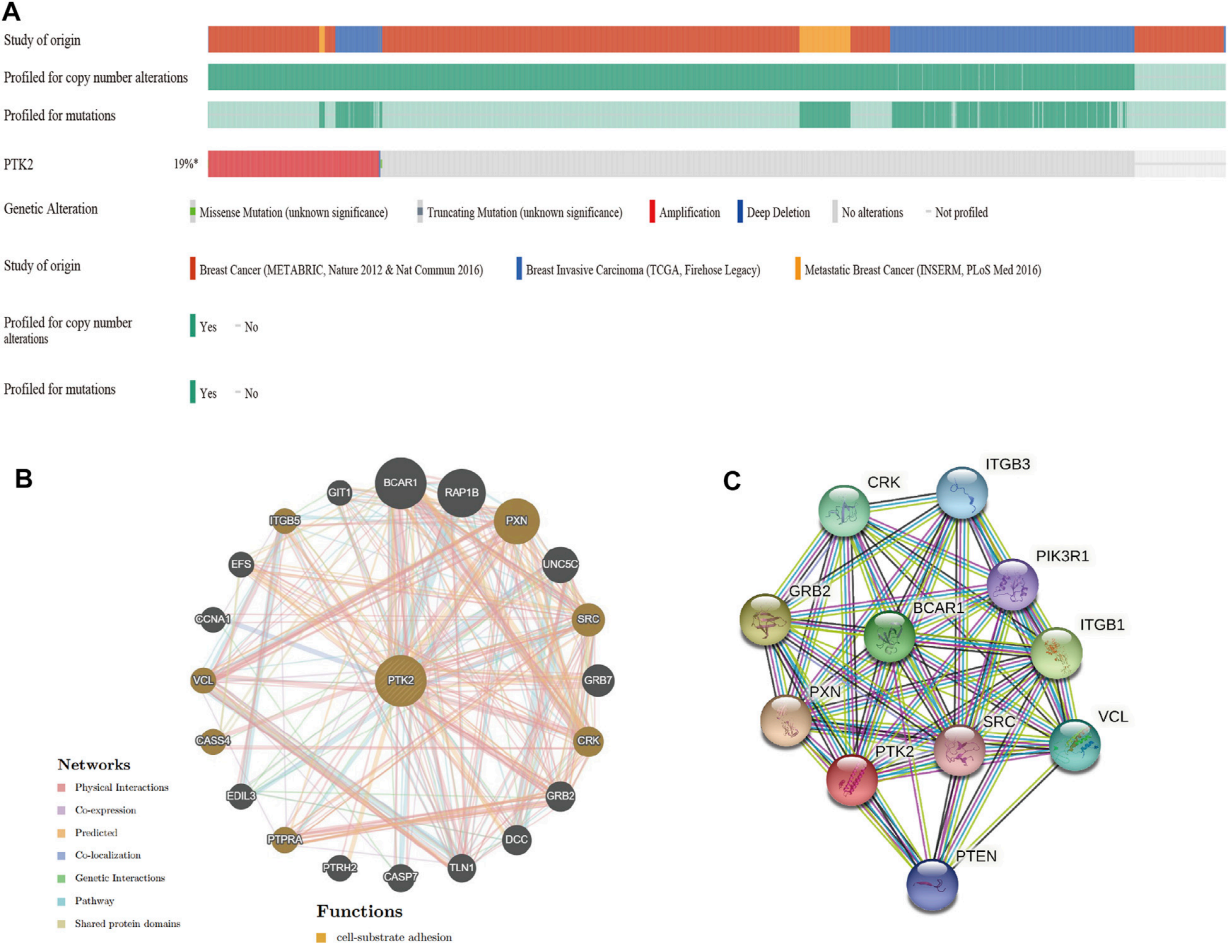
Genomic alterations of PTK2 in breast cancer. **(A)** Overview of genomic alterations of the PTK2 in breast cancer using OncoPrint schematic. **(B)** Gene–gene interaction network of PTK2 was constructed using GeneMania. **(C)** PPI network of PTK2 was generated using STRING.

Given that the genomic alteration of PTK2 is pathogenic, the cBioPortal database was used to investigate the genetic alteration of PTK2. PTK2 alterations were found in 28 of 360 patients (19%) using the database (Figure 4A). Among these patients, PTK2 gene alterations were mainly amplified in breast cancer, invasive breast cancer, and metastatic breast cancer (Supplementary Figure S2A). In addition, we assessed the association of PTK2 gene alterations with the survival of breast cancer patients. There was a significant relationship between overall survival (OS) and relapse-free survival (RFS) of breast cancer patients with PTK2 gene alterations, but not with disease-free survival (Supplementary Figure S2B). In addition, the gene–gene interaction network for PTK2 and the altered neighboring genes was constructed using GeneMANIA. The results showed that the 20 most frequently altered genes were closely correlated with PTK2. Functional analysis suggested that these genes were significantly associated with cell–substrate adhesion (Figure 4B). A protein–protein interaction network of PTK2 was generated using the STRING database, and the strongest interactions were found with the PXN, GRB2, ITGB1, BCAR1, CRK, VCL, ITGB3, PTEN, PIK3R1, and SRC proteins (Figure 4C).

### Enrichment analysis of PTK2 gene co-expression network in breast cancer

**FIGURE 5:**
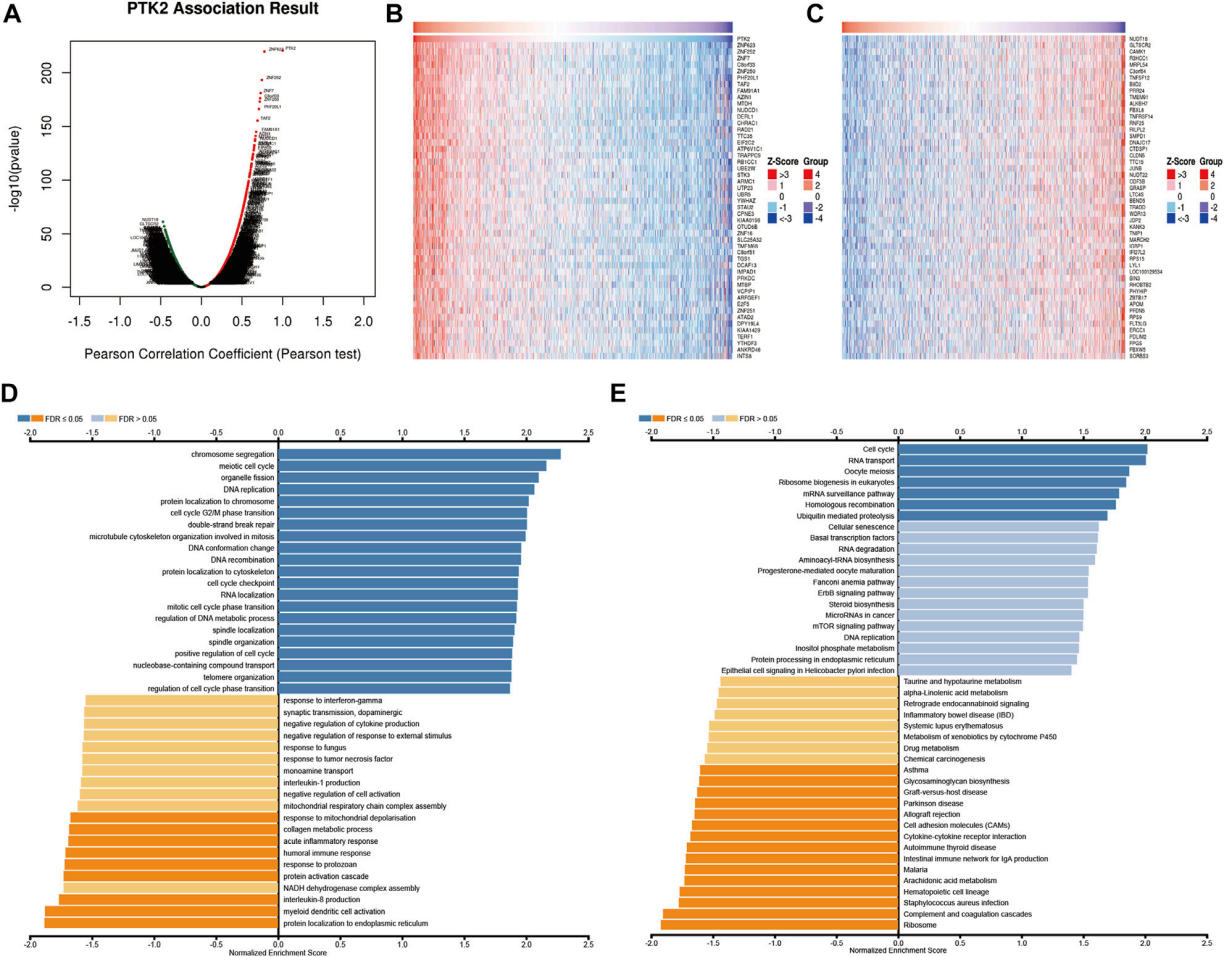
PTK2 co-expressed genes and functional enrichment analysis (LinkedOmics). **(A)** Volcano plot of co-expressed profiling of PTK2 in breast cancer; red (positive) and green (negative). **(B,C)** Heat maps showing the top 50 positively and 50 negatively correlated genes with PTK2 in breast cancer. The BP **(D)** and KEGG **(E)** of PTK2 were analyzed by LinkedOmics.

To understand the biological significance of PTK2 in breast cancer, we analyzed the positively and negatively correlated co-expressed genes of PTK2 in breast cancer utilizing the LinkedOmics database. As shown in Figure 5A, we found that 5,584 genes were positively correlated with PTK2 and that 7,984 genes were negatively correlated with PTK2 (FDR <0.05). The top 50 significant genes with positive PTK2 correlations (Figure 5B) and negative correlations (Figure 5C) are shown in the heatmap. As shown in Figure 5D, Gene Ontology analysis carried out by GSEA revealed that PTK2 co-expressed genes were mainly involved in the following biological processes (BPs): chromosome segregation, meiotic cell cycle, organelle fission, DNA replication, protein localization to chromosome, protein localization to endoplasmic reticulum, myeloid dendritic cell activation, interleukin-8 production, NADH dehydrogenase complex assembly, and the protein activation cascade. In terms of cellular components (CCs), the chromosomal region, condensed chromosome, spindle, replication fork, nuclear periphery, extracellular matrix, protein–lipid complex, collagen trimer, respiratory chain, and NADH dehydrogenase complex were the most prominent (Supplementary Figure S3A). In terms of molecular functions (MFs), histone binding, single-stranded DNA binding, double-stranded RNA binding, ATPase activity, helicase activity, extracellular matrix structural constituent, structural constituent of ribosome, oxidoreductase activity, acting on peroxide as acceptor, serine hydrolase activity, and antioxidant activity were the most significant (Supplementary Figure S3B). The KEGG pathway analysis showed that PTK2 and co-expressed genes were significantly enriched in homologous recombination, mRNA surveillance pathway, ribosome biogenesis in eukaryotes, oocyte meiosis, RNA transport, cell cycle, ribosome, complement, and coagulation cascades, *Staphylococcus aureus* infection, hematopoietic cell lineage, and arachidonic acid metabolism (Figure 5E).

### Correlation between PTK2 expression and immune signatures in breast cancer

**FIGURE 6:**
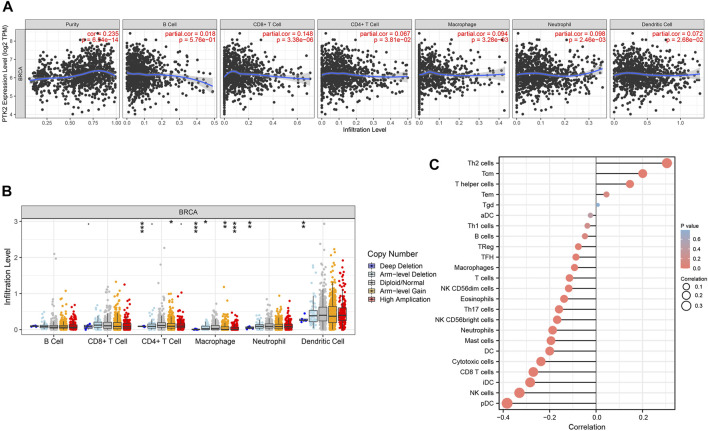
Correlations of PTK2 expression with immune infiltration level in breast cancer. **(A)** PTK2 is significantly associated with tumor purity and is correlated with the infiltration of different immune cells using the TIMER database. **(B)** PTK2 CNV affects the infiltrating levels of CD4^+^ T cells, macrophages, neutrophils, and dendritic cells in breast cancer. **(C)** PTK2 expression has a significant correlation with the infiltration of immune cells in breast cancer using the ssGSEA analyses. *p < 0.05; **p < 0.01; ***p < 0.001.

Tumor-infiltrating lymphocytes have been considered to be an independent predictor of cancer prognosis. Consequently, to deepen the understanding of PTK2 crosstalk with the immune response, we used the TIMER database to validate the relationship between PTK2 expression and diverse immune signatures in breast cancer. As shown in Figure 6A, PTK2 was positively correlated with infiltrating levels of CD8^+^ T cells, CD4^+^ T cells, macrophages, neutrophils, and dendritic cells. However, PTK2 CNV was also found to be significantly correlated with the infiltration levels of CD4^+^ T cells, macrophages, neutrophils, and dendritic cells (Figure 6B). We estimated the correlation between PTK2 and immune infiltration using ssGSEA. Notably, PTK2 was correlated with the infiltration levels of most immune cells (Figure 6C). In addition, PTK2 was significantly correlated with the gene markers of B cells, T cells, CD8^+^ T cells, monocytes, tumor-associated macrophages (TAMs), M1 macrophages, M2 macrophages, neutrophils, natural killer (NK) cells, and dendritic cells (Table 1).

**TABLE 1:** Correlation analysis between PTK2 and gene markers of immune cells in breast cancer by TIMER.

Description	Gene markers	COAD			
		None		Purity	
		Cor	p	Cor	p
B cell	CD19	−0.08839	0.003348	−0.09285	0.003375
	CD79A	−0.10338	0.000595	−0.10656	0.000761
T cell (general)	CD3D	−0.13937	3.49E-06	−0.13548	1.80E-05
	CD3E	−0.10682	0.000387	−0.10027	0.001541
	CD2	−0.06981	0.020584	−0.06365	0.044733
CD8+T cell	CD8A	−0.05036	0.095041	−0.03971	0.210697
	CD8B	−0.09437	0.001728	−0.08105	0.010539
	CD86	0.016308	0.588989	0.028653	0.366594
	CSF1R	−0.11389	0.000154	−0.11095	0.000455
TAM	CCL2	−0.00687	0.819946	−0.00354	0.911194
	CD68	−0.01119	0.710859	−0.00157	0.960514
	IL10	0.07919	0.008599	0.076086	0.016374
M1 macrophage	IRF5	0.052933	0.07929	0.047974	0.130474
	PTGS2	−0.00977	0.746287	−0.00586	0.853481
	NOS2	0.028841	0.339251	0.03489	0.271541
M2 macrophage	CD163	0.081293	0.006985	0.094037	0.002987
	VSIG4	−0.03805	0.207337	−0.03162	0.319007
	MS4A4A	0.004292	0.88692	0.013319	0.67476
Neutrophils	CEACAM8	0.018107	0.548559	0.018593	0.558018
	ITGAM	−0.05845	0.052638	−0.05024	0.113223
	CCR7	−0.05281	0.080001	−0.04714	0.137308
Natural killer cell	KIR2DL1	−0.0171	0.571048	−0.01495	0.637722
	KIR2DL3	0.002502	0.933931	−0.00878	0.78202
	KIR2DL4	−0.00051	0.986422	0.016328	0.606951
	KIR3DL1	−0.01973	0.513238	−0.01226	0.699244
	KIR3DL2	−0.01648	0.585005	−0.01677	0.59724
	KIR3DL3	0.006761	0.822772	0.006832	0.829587
	KIR2DS4	−0.01138	0.706045	0.00992	0.754646
Dendritic cell	HLA-DPB1	−0.26164	1.13E-18	−0.25584	2.47E-16
	HLA-DQB1	−0.16645	2.81E-08	−0.15597	7.66E-07
	HLA-DRA	−0.09266	0.002096	−0.08427	0.007822
	HLA-DPA1	−0.11428	0.000146	−0.1031	0.001126

### Methylation analysis of PTK2 in breast cancer

**FIGURE 7:**
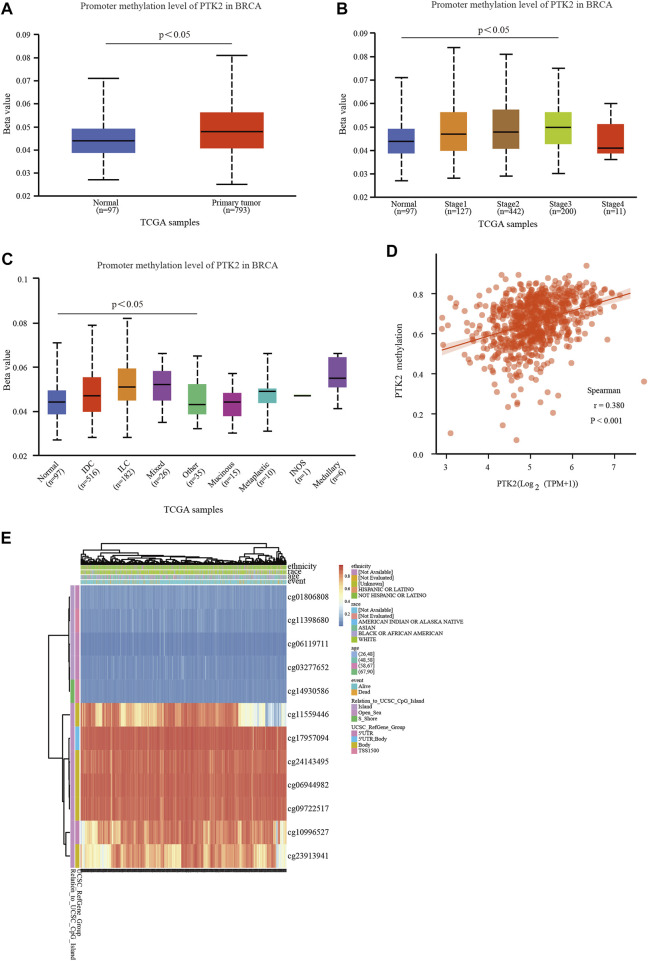
DNA methylation level and its prognostic value of PTK2 in breast cancer. **(A)** Promoter methylation of PTK2 in normal and cancer tissues from TCGA-breast cancer data. **(B,C)** Promoter methylation level of PTK2 in breast cancer of different tumor stages and tumor types using the UALCAN database. **(D)** Correlation between PTK2 methylation and its expression level. **(E)** Heat map of DNA methylation at CpG sites in the PTK2 gene using the MethSurv database.

To further explore the potential role of PTK2 in breast cancer tumorigenesis, the methylation level of PTK2 was assessed in breast cancer. As shown in Figure 7A, the methylation level of the PTK2 gene in breast cancer was higher than that in normal tissue. In addition, it was also found that the methylation levels of breast cancer tumor stages 1–3 were higher than those of normal tissue (Figure 7B), and invasive duct carcinoma, invasive lobular carcinoma, and mixed breast cancer showed higher methylation expression than normal breast tissue (Figure 7C). We then analyzed the correlation between PTK2 expression and methylation levels. As shown in Figure 7D, PTK2 mRNA expression was positively correlated with DNA methylation. The MethSurv database was used to further validate the higher methylation level in breast cancer. As shown in Figure 7E, PTK2 has 12 methylation sites, of which cg23913941, cg10996527, cg09722517, cg06944982, cg24143495, cg17957094, and cg11559446 have the highest methylation level. Among 12 CpG sites of PTK2, cg11398680, cg11559446, cg23913941, and cg24143495 were associated with poor prognosis in breast cancer patients (Table 2).

**TABLE 2:** Significant prognostic values of CpG in PTK2.

Name	UCSC_RefGene_group	Relation_to_UCSC_CpG_island	HR	CI	*p*-value
cg01806808	5'UTR	Island	0.741	(0.44; 1.249)	0.260258
cg03277652	5'UTR	Island	0.67	(0.426; 1.055)	0.083719
cg06119711	5'UTR	Island	0.795	(0.518; 1.22)	0.29328
cg06944982	Body	Open_Sea	0.855	(0.537; 1.359)	0.507044
cg09722517	Body	Open_Sea	1.491	(0.895; 2.484)	0.124797
cg10996527	5'UTR	Open_Sea	0.751	(0.464; 1.215)	0.243985
cg11398680	TSS1500	Island	0.586	(0.381; 0.902)	0.01517
cg11559446	Body	Open_Sea	0.371	(0.241; 0.572)	7.17E-06
cg14930586	TSS1500	S_Shore	1.453	(0.986; 2.141)	0.058642
cg17957094	5'UTR; Body	Open_Sea	0.872	(0.585; 1.3)	0.501601
cg23913941	Body	Open_Sea	0.608	(0.41; 0.9)	0.01291
cg24143495	Body	Open_Sea	1.783	(1.014; 3.135)	0.044609

### PTK2-related ceRNA network construction in breast cancer

**FIGURE 8:**
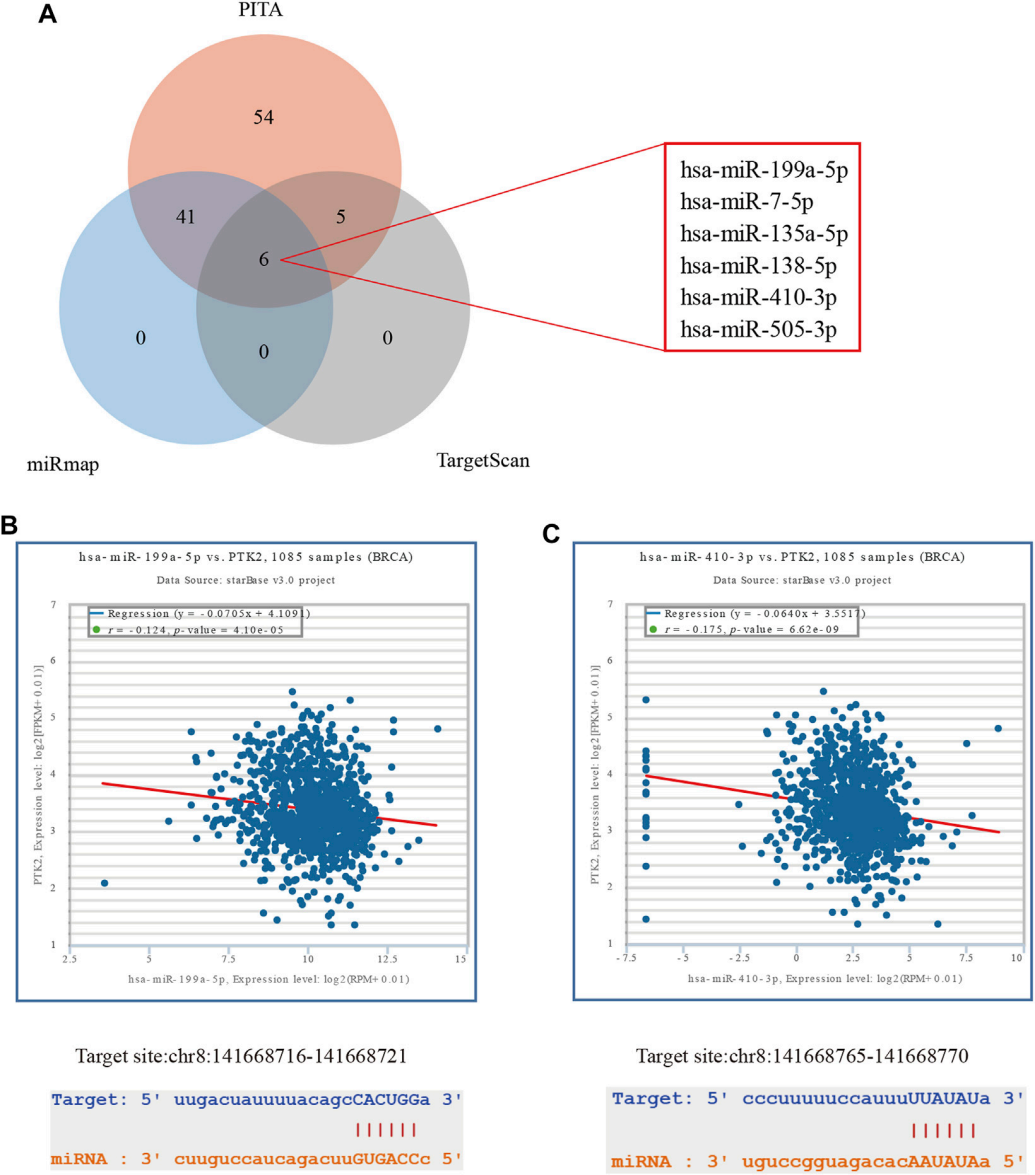
Predicting miRNAs targeting PTK2 in breast cancer. **(A)** Prediction results of PTK2 targets in the three databases PITA, miRmap, and TargetScan are shown by a Venn diagram. **(B)** starBase online database was used to analyze the correlation between PTK2 and target miRNAs. **(C)** TargetScan predicts the potential binding site of PTK2 to the target miRNAs.

**FIGURE 9:**
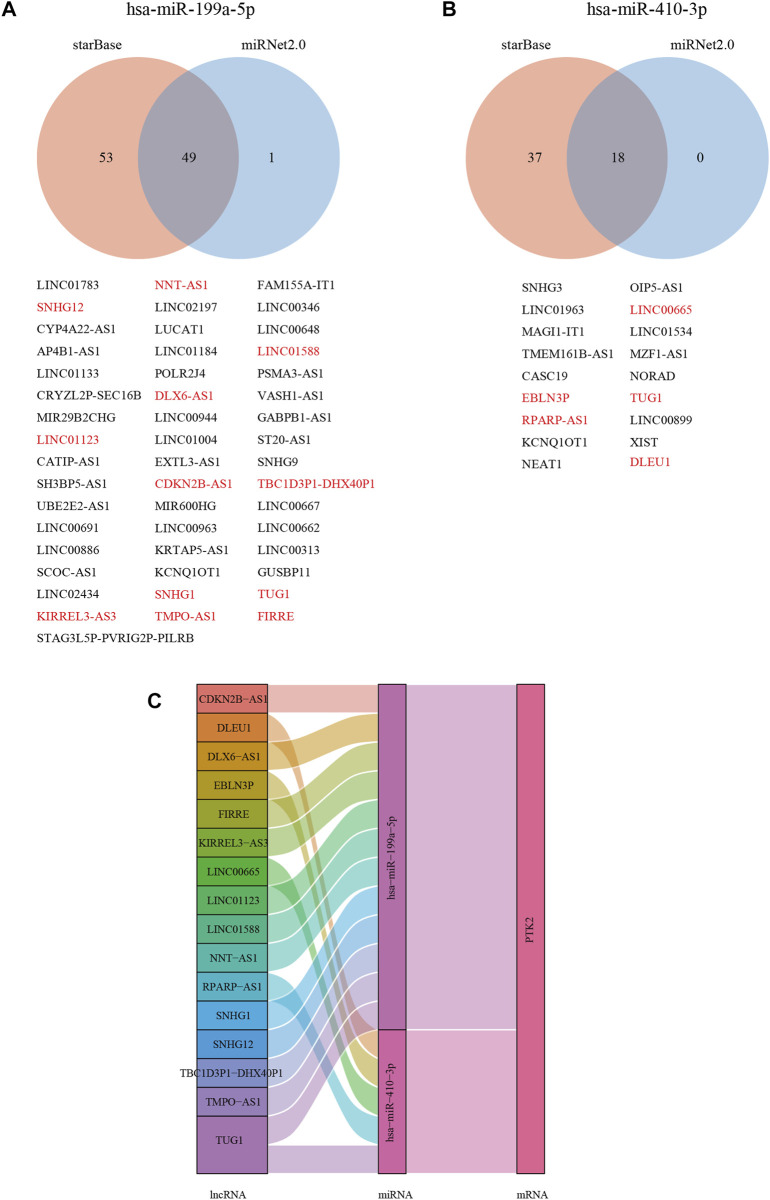
Construction of lncRNA and ceRNA network in breast cancer. The Venn diagram shows the target lncRNA of hsa-miR-199a-5p **(A)** and hsa-miR-410-3p **(B)**. Red (negative correlation with miRNAs). **(C)** Hypothesis of the lncRNA–miRNA–mRNA (PTK2) regulatory network shown by a Sankey diagram.

With increasing evidence that the lncRNA–miRNA–mRNA ceRNA network plays a critical role in multiple human cancers, we sought to analyze and construct a breast cancer ceRNA network involving PTK2. A total of 106, 47, and 11 PTK2 target miRNAs were analyzed and predicted using three respective databases: PITA, miRmap, and TargetScan. The predicted results of PTK2 target miRNAs in PITA, miRmap, and TargetScan software are shown in the Venn diagram (Figure 8A). A total of six target miRNAs were found in the database: hsa-miR-199a-5p, hsa-miR-7-5p, hsa-miR-135a-5p, hsa-miR-138–5p, hsa-miR-410–3p, and hsa-miR-505–3p. In addition, we also analyzed the correlation between target miRNA and PTK2 expression and screened out a miRNA that was more suitable for ceRNA conditions. The expression levels of hsa-miR-199a-5p (*r* = −0.124, *p* < 0.0001) and hsa-miR-410–3p (*r* = −0.175, *p* < 0.0001) were negatively correlated with PTK2 expression (Figure 8B). We also used TargetScan software to predict the potential binding sites of PTK2 and target miRNAs (Figure 8B). Moreover, we further predicted lncRNAs that might bind to the targets hsa-miR-199a-5p and hsa-miR-410–3p using the miRNet2.0 and starBase online databases and presented them in a Venn diagram (Figures 9A,B). The regulatory network of lncRNA–miRNA (hsa-miR-199a-5p)–mRNA (PTK2) and lncRNA–miRNA (hsa-miR-410–3p)–mRNA (PTK2) is shown in Figure 9C.

### Effects of PTK2 knockdown on the proliferation and migration in breast cancer cells

**FIGURE 10:**
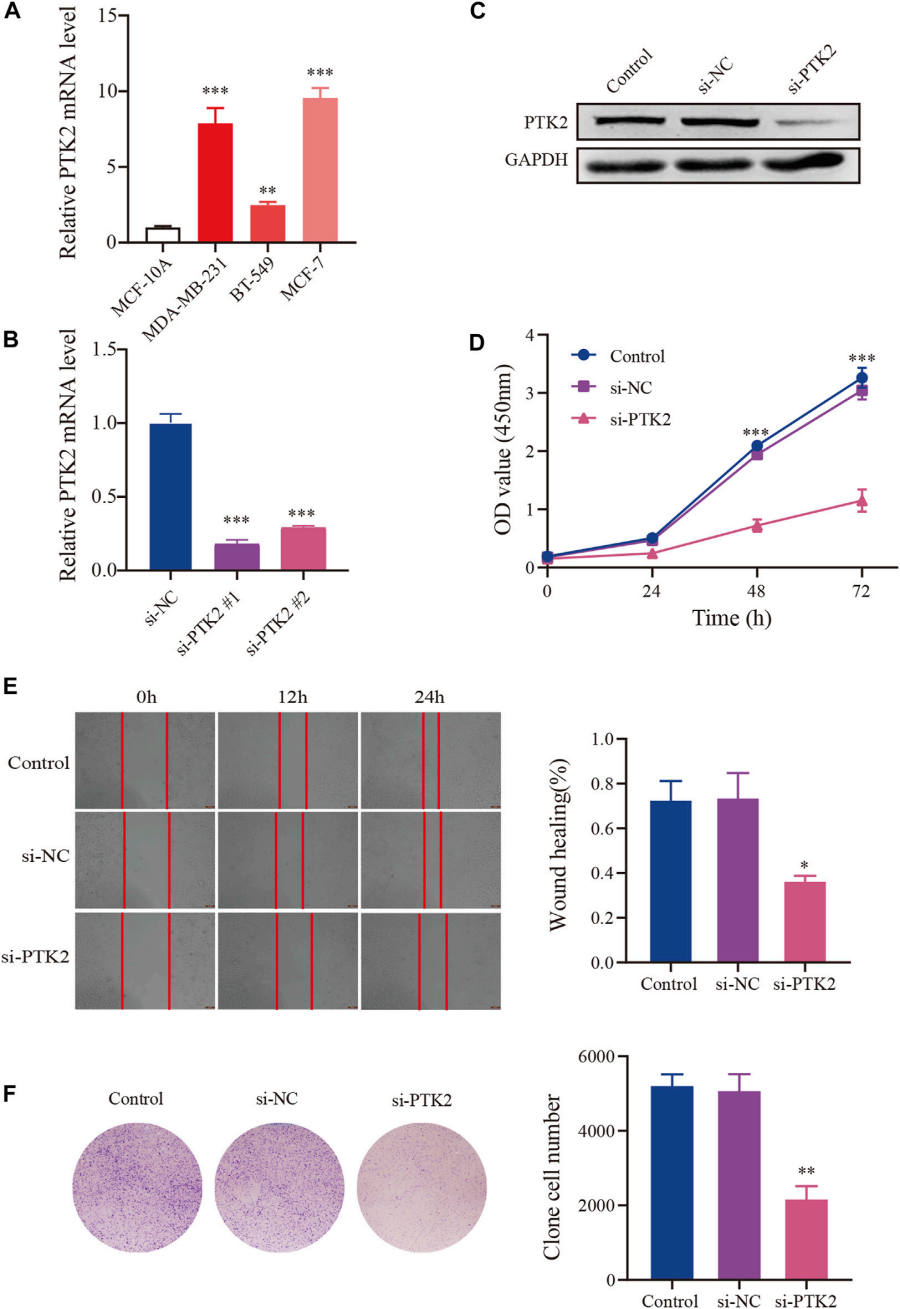
Knockdown of NR2F6 inhibits malignant phenotypes in MCF7 cells. **(A)** PTK2 mRNA expression level in breast cancer cell lines. **(B)** Knockdown efficacy of PTK2 siRNAs in MCF7 cells detected by RT–qPCR. **(C)** PTK2 protein expression level of MCF7 cells in control, si-NC, and si-PTK2 groups. **(D)** CCK8 was performed to estimate the effects of PTK2 knockdown on MCF7 cell proliferation. **(E)** Colony forming ability of MCF7 cells transfected with si-PTK2. **(F)** Wound healing assay after transfection with PTK2 siRNA or control siRNA in MCF7 cells. Quantitative data are indicated as mean ± SEM (*n* = 3). *p < 0.05, **p < 0.01, and ***p < 0.001 vs. si-NC and control groups.

Next, we performed in vitro experiments to explore the effects of PTK2 on the biological behavior of breast cancer. We first examined PTK2 expression in breast cancer cell lines (MCF-7, BT-549, and MDA-MB-231) and the normal human mammary epithelial cell line, MCF-10A. The results revealed that PTK2 mRNA expression level was significantly increased in MCF-7, BT-549, and MDA-MB-231 compared with MCF-10A. Subsequently, MCF-7 was transfected with siRNA (si-PTK2 #1, si-PTK2 #2) to knockdown PTK2 expression. As shown in Figure 10B, the PTK2 mRNA level in MCF-7 cells was significantly decreased after transfection with si-PTK2 #1, with si-PTK2 #1 knockdown efficiency being the highest. Meanwhile, the PTK2 protein level in MCF-7 cells was significantly decreased after transfection with si-PTK2 #1 (Figure 10C). CCK8 assay was used to assess the effect of PTK2 on the proliferation of MCF-7 cells, and results suggested that cell proliferation was significantly decreased in the si-PTK2 group (Figure 10D). Colony formation assay showed that knockdown of PTK2 suppressed the clone-forming capability of MCF-7 cells (Figure 10E). Wound healing assay showed that the cell migration ability of the si-PTK2 group was significantly reduced compared with that of the si-NC and control groups (Figure 10F). Collectively, these results indicated that the knockdown of PTK2 could suppress proliferation and migration in MCF7 cells.

## Discussion

Breast cancer remains the most common cancer among women worldwide with high rates of recurrence and disease progression (Jemal et al., 2012; Anastasiadi et al., 2017). Therefore, there is an urgent need to develop reliable diagnostic, prognostic, and therapeutic biomarkers for breast cancer. In this study, we explored the prognostic value and biological functions of PTK2 in breast cancer using various public databases. A growing amount of research suggests that PTK2 can promote cell proliferation, motility, adipogenesis, metastasis, glucose consumption, and glutathione amino-dependence (Zhang and Hochwald, 2014). PTK2 is considered a positive regulator in the progression and metastasis of various types of cancer. Overexpression of PTK2 has been described in a diverse assortment of human tumors, including breast cancer (Sethuraman et al., 2016), hepatocellular carcinoma, and head and neck cell carcinoma (Fan et al., 2019).

PTK2 is highly expressed in diverse cell lines and different tissues of many human tumors (de Ruiter and Willems, 2016; Skinner et al., 2016). In addition, PTK2 overexpression is associated with poor prognosis in several tumor types (Miyasaka et al., 2001; Fujii et al., 2004; Itoh et al., 2004). In this study, we used bioinformatics analysis of the TIMER, UALCAN, and TCGA public databases and found that PTK2 expression levels in breast cancer tissues were higher than those in normal breast tissues. Analysis of the TIMER database found that PTK2 was highly expressed in 13 cancers, and the expression of PTK2 in breast cancer samples was significantly increased. In addition, to better understand whether PTK2 could affect the progression of breast cancer, we explored the association between PTK2 and clinicopathological characteristics in breast cancer. High PTK2 expression was associated with cancer stage, tumor histology, TP53-mutant, and meno-menopausal pause status. Next, Kaplan–Meier survival curves and PrognoScan analysis showed that PTK2 was highly expressed in breast cancer and was related to poor prognosis. These lines of evidence suggest that PTK2 has a crucial role in tumor occurrence and development.

To unravel the biological functions of PTK2, the LinkedOmics database was used to perform co-expression analysis and functional enrichment in breast cancer. Through GO and KEGG pathway analyses of 100 genes related to PTK2, PTK2 co-expression was found to be mainly related to chromosome segregation, meiotic cell cycle, organelle fission, and DNA replication. KEGG pathway analysis showed that the PTK2 co-expression was mainly related to the cell cycle signaling pathway. Mutations and/or dysregulation in DNA methylation will affect tumor progression, and Fan et al. (2019) found that the methylation level of the PTK2 promoter regulated the expression level of PTK2 in hepatocellular carcinoma. Next, we explored the DNA methylation level of PTK2 in breast cancer. Our results showed that PTK2 overexpression might be related to PTK2 hypomethylation and that PTK2 mRNA expression was positively correlated with the level of methylation. In addition, we found that PTK2 methylation at certain CpG sites was correlated with poor prognosis in breast cancer patients, indicating that methylation levels of PTK2 act as a powerful prognostic biomarker.

An increasing number of studies have shown that lncRNAs act as competing endogenous RNAs (ceRNAs) by decoying miRNAs to regulate mRNA expression. As expected, perturbation of these interactive networks leads to various diseases, including cancer. Zhang et al. (2020) found that miR-520d-5p overexpression can significantly inhibit the expression of PTK2, whereas downregulation of miR-520d-5p can promote the expression of PTK2 in clear cell renal cell carcinoma. Thus, we constructed a ceRNA network based on PTK2 expression. In this study, three databases were used to predict upstream miRNAs of PTK2, but only two miRNAs (hsa-miR-199a-5p and hsa-miR-410–3p) were significantly negatively correlated with PTK2 in breast cancer. Ahmadi et al. reported that hsa-miR-199a-5p had a causative effect on tumorigenesis in lung cancer and possibly other cancer types. Qi et al. (2021) reported that hsa-MIR-410–3p expression was decreased in head and neck squamous cell carcinomas. Next, we predicted the upstream lncRNAs of these key miRNAs. A total of 16 lncRNAs (CDKN2B-AS1, DLEU1, DLX6-AS1, EBLN3P, FIRRE, KIRRIL3-AS3, LINC00665, LINC01123, LINC01588, NNT-AS1, RPAPR-AS1, SNHG12, TBC1D3P1-DHX40P1, TMPO-AS1, and TUG1) were identified as key lncRNAs. It was recently shown that the expression level of lncRNA CDKN2B-AS1 was notably upregulated in lung cancer, and the overexpression of CDKN2B-AS1 could promote tumor cell proliferation and invasion (Wang et al., 2020). DLEU1 was upregulated in non-small cell lung cancer tissues and promoted the proliferation, migration, and invasion of tumor cells (Zhang et al., 2019). These lncRNAs, miRNAs, and genes form the ceRNA regulatory network, which is involved in the development of breast cancer. Taken together, PTK2 upregulation is strongly associated with poor prognosis, clinicopathological features, and methylation. In addition, we established a ceRNA network through the database. Therefore, this study provides ideas for further research on breast cancer treatment. The current study is an initial part of a larger study. We will perform further validation and experiments with independent datasets in the future.

In conclusion, our study highlighted the value of PTK2 as a potential novel prognostic biomarker for breast cancer. PTK2 knockdown could suppress the proliferation and migration ability of MCF7 cells. Moreover, we explored the underlying evidence indicating that hepcidin might regulate the cell cycle in breast cancer patients. The construction of a ceRNA network of PTK2 indicates that PTK2 may be involved in a variety of molecular mechanisms in breast cancer.

## Data Availability

The original contributions presented in the study are included in the article/Supplementary Material; further inquiries can be directed to the corresponding author.

## References

[B1] Anastasiadi Z. Lianos G. D. Ignatiadou E. Harissis H. V. Mitsis M. (2017). Breast cancer in young women: an overview. Updat. Surg. 69 (3), 313–317. 10.1007/s13304-017-0424-1 28260181

[B2] Batista S. Maniati E. Reynolds L. E. Tavora B. Lees D. M. Fernandez I. (2014). Haematopoietic focal adhesion kinase deficiency alters haematopoietic homeostasis to drive tumour metastasis. Nat. Commun. 5, 5054. 10.1038/ncomms6054 25270220

[B3] Chandran U. R. Medvedeva O. P. Barmada M. M. Blood P. D. Chakka A. Luthra S. (2016). TCGA expedition: A data acquisition and management system for TCGA data. PLoS One 11 (10), e0165395. 10.1371/journal.pone.0165395 27788220 PMC5082933

[B4] Chandrashekar D. S. Bashel B. Balasubramanya S. A. H. Creighton C. J. Ponce-Rodriguez I. Chakravarthi B. (2017). UALCAN: A portal for facilitating tumor subgroup gene expression and survival analyses. Neoplasia 19 (8), 649–658. 10.1016/j.neo.2017.05.002 28732212 PMC5516091

[B5] de Ruiter E. J. Willems S. M. (2016). PTK2/FAK: a new predictive biomarker for response to radiotherapy in head and neck squamous cell carcinoma. Ann. Transl. Med. 4 (1), S44. 10.21037/atm.2016.10.19 27868012 PMC5104593

[B6] Fan Z. Duan J. Wang L. Xiao S. Li L. Yan X. (2019). PTK2 promotes cancer stem cell traits in hepatocellular carcinoma by activating Wnt/β-catenin signaling. Cancer Lett. 450, 132–143. 10.1016/j.canlet.2019.02.040 30849480

[B7] Fang J. Ge X. Xu W. Xie J. Qin Z. Shi L. (2020). Bioinformatics analysis of the prognosis and biological significance of HMGB1, HMGB2, and HMGB3 in gastric cancer. J. Cell. Physiol. 235 (4), 3438–3446. 10.1002/jcp.29233 31621076 PMC13482706

[B8] Fujii T. Koshikawa K. Nomoto S. Okochi O. Kaneko T. Inoue S. (2004). Focal adhesion kinase is overexpressed in hepatocellular carcinoma and can be served as an independent prognostic factor. J. Hepatol. 41 (1), 104–111. 10.1016/j.jhep.2004.03.029 15246215

[B9] Itoh S. Maeda T. Shimada M. Aishima S. Shirabe K. Tanaka S. (2004). Role of expression of focal adhesion kinase in progression of hepatocellular carcinoma. Clin. Cancer Res. 10 (8), 2812–2817. 10.1158/1078-0432.ccr-1046-03 15102689

[B10] Jemal A. Bray F. Forman D. O'Brien M. Ferlay J. Center M. (2012). Cancer burden in Africa and opportunities for prevention. Cancer 118 (18), 4372–4384. 10.1002/cncr.27410 22252462

[B11] Li T. Fan J. Wang B. Traugh N. Chen Q. Liu J. S. (2017). TIMER: A web server for comprehensive analysis of tumor-infiltrating immune cells. Cancer Res. 77 (21), e108–e110. 10.1158/0008-5472.CAN-17-0307 29092952 PMC6042652

[B12] McLean G. W. Carragher N. O. Avizienyte E. Evans J. Brunton V. G. Frame M. C. (2005). The role of focal-adhesion kinase in cancer - a new therapeutic opportunity. Nat. Rev. Cancer 5 (7), 505–515. 10.1038/nrc1647 16069815

[B13] Miyasaka Y. Enomoto N. Nagayama K. Izumi N. Marumo F. Watanabe M. (2001). Analysis of differentially expressed genes in human hepatocellular carcinoma using suppression subtractive hybridization. Br. J. Cancer 85 (2), 228–234. 10.1054/bjoc.2001.1901 11461082 PMC2364030

[B14] Mizuno H. Kitada K. Nakai K. Sarai A. (2009). PrognoScan: a new database for meta-analysis of the prognostic value of genes. BMC Med. Genomics 2, 18. 10.1186/1755-8794-2-18 19393097 PMC2689870

[B15] Modhukur V. Iljasenko T. Metsalu T. Lokk K. Laisk-Podar T. Vilo J. (2018). MethSurv: a web tool to perform multivariable survival analysis using DNA methylation data. Epigenomics 10 (3), 277–288. 10.2217/epi-2017-0118 29264942

[B16] Montojo J. Zuberi K. Rodriguez H. Bader G. D. Morris Q. (2014). GeneMANIA: Fast gene network construction and function prediction for Cytoscape. F1000Res. 3, 153. 10.12688/f1000research.4572.1 25254104 PMC4168749

[B17] Qi C. L. Sheng J. F. Huang M. L. Zou Y. Wang Y. P. Wang F. (2021). Integrated analysis of deregulation microRNA expression in head and neck squamous cell carcinoma. Med. Baltim. 100 (6), e24618. 10.1097/MD.0000000000024618 PMC7886409 33578572

[B18] Sethuraman A. Brown M. Seagroves T. N. Wu Z. H. Pfeffer L. M. Fan M. (2016). SMARCE1 regulates metastatic potential of breast cancer cells through the HIF1A/PTK2 pathway. Breast Cancer Res. 18 (1), 81. 10.1186/s13058-016-0738-9 27495308 PMC4974701

[B19] Sieg D. J. Hauck C. R. Ilic D. Klingbeil C. K. Schaefer E. Damsky C. H. (2000). FAK integrates growth-factor and integrin signals to promote cell migration. Nat. Cell Biol. 2 (5), 249–256. 10.1038/35010517 10806474

[B20] Skinner H. D. Giri U. Yang L. Woo S. H. Story M. D. Pickering C. R. (2016). Proteomic profiling identifies PTK2/FAK as a driver of radioresistance in HPV-negative head and neck cancer. Clin. Cancer Res. 22 (18), 4643–4650. 10.1158/1078-0432.CCR-15-2785 27036135 PMC5061056

[B21] Sulzmaier F. J. Jean C. Schlaepfer D. D. (2014). FAK in cancer: mechanistic findings and clinical applications. Nat. Rev. Cancer 14 (9), 598–610. 10.1038/nrc3792 25098269 PMC4365862

[B22] Thul P. J. Lindskog C. (2018). The human protein atlas: A spatial map of the human proteome. Protein Sci. 27 (1), 233–244. 10.1002/pro.3307 28940711 PMC5734309

[B23] Vasaikar S. V. Straub P. Wang J. Zhang B. (2018). LinkedOmics: analyzing multi-omics data within and across 32 cancer types. Nucleic Acids Res. 46 (D1), D956–D963. 10.1093/nar/gkx1090 29136207 PMC5753188

[B24] von Mering C. Huynen M. Jaeggi D. Schmidt S. Bork P. Snel B. (2003). STRING: a database of predicted functional associations between proteins. Nucleic Acids Res. 31 (1), 258–261. 10.1093/nar/gkg034 12519996 PMC165481

[B25] Wang G. Xu G. Wang W. (2020). Long noncoding RNA CDKN2B-AS1 facilitates lung cancer development through regulating miR-378b/NR2C2. Onco. Targets. Ther. 13, 10641–10649. 10.2147/OTT.S261973 33116641 PMC7585785

[B26] Weisser M. Yeh R. F. Duchateau-Nguyen G. Palermo G. Nguyen T. Q. Shi X. (2014). PTK2 expression and immunochemotherapy outcome in chronic lymphocytic leukemia. Blood 124 (3), 420–425. 10.1182/blood-2013-12-538975 24916506

[B27] Zhang J. Hochwald S. N. (2014). The role of FAK in tumor metabolism and therapy. Pharmacol. Ther. 142 (2), 154–163. 10.1016/j.pharmthera.2013.12.003 24333503 PMC6349254

[B28] Zhang L. Liu F. Fu Y. Chen X. Zhang D. (2020). MiR-520d-5p functions as a tumor-suppressor gene in cervical cancer through targeting PTK2. Life Sci. 254, 117558. 10.1016/j.lfs.2020.117558 32198053

[B29] Zhang S. Guan Y. Liu X. Ju M. Zhang Q. (2019). Long non-coding RNA DLEU1 exerts an oncogenic function in non-small cell lung cancer. Biomed. Pharmacother. 109, 985–990. 10.1016/j.biopha.2018.10.175 30551552

[B30] Zhao X. Guan J. L. (2011). Focal adhesion kinase and its signaling pathways in cell migration and angiogenesis. Adv. Drug Deliv. Rev. 63 (8), 610–615. 10.1016/j.addr.2010.11.001 21118706 PMC3132829

