# Supplementary material for: Identification of PTK2 as an adverse prognostic biomarker in breast cancer by integrated bioinformatics and experimental analyses
Source: Front Mol Biosci. 2022 Dec 1;9:984564. doi: 10.3389/fmolb.2022.984564 (PMC9751198; doi:10.3389/fmolb.2022.984564)

Supplementary Material

**Figure S1. Expression of PTK2 in human normal tissues and cell lines.** (A) PTK2 mRNA expression in different breast cancer cell lines. (B) PTK2 mRNA expression in kinds of normal tissues from the GTEx project. (C) The expression level of PTK2 in different breast cells.


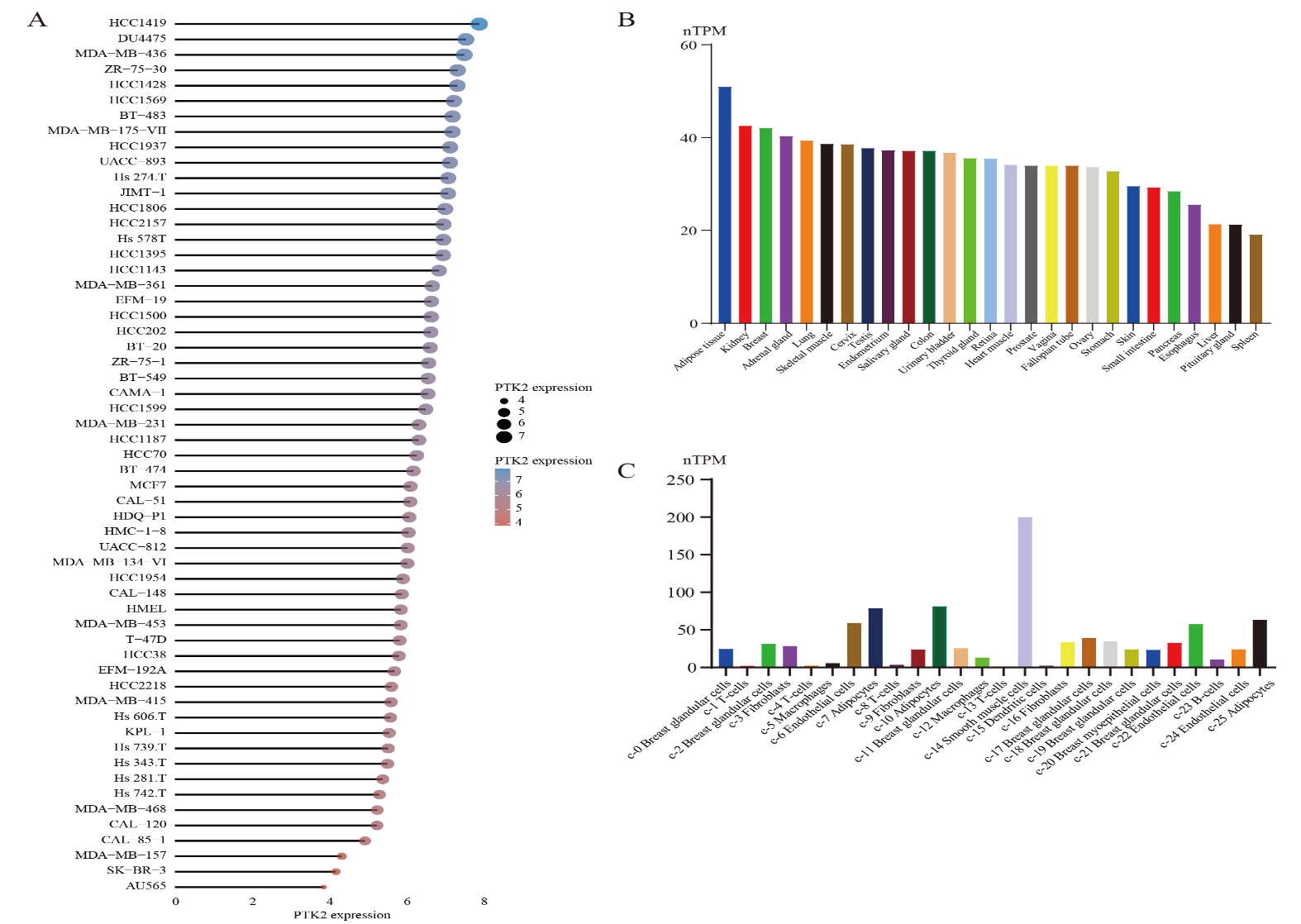


**Figure S2.** **Gene alterations in PTK2 in breast cancer.** (A) Frequency of gene alterations in PTK2 in different types of breast cancer. (B) Kaplan‐Meier survival curves were used to analyze the correlation between gene alterations in PTK2 and OS, RFS, and DFs of breast cancer patients.


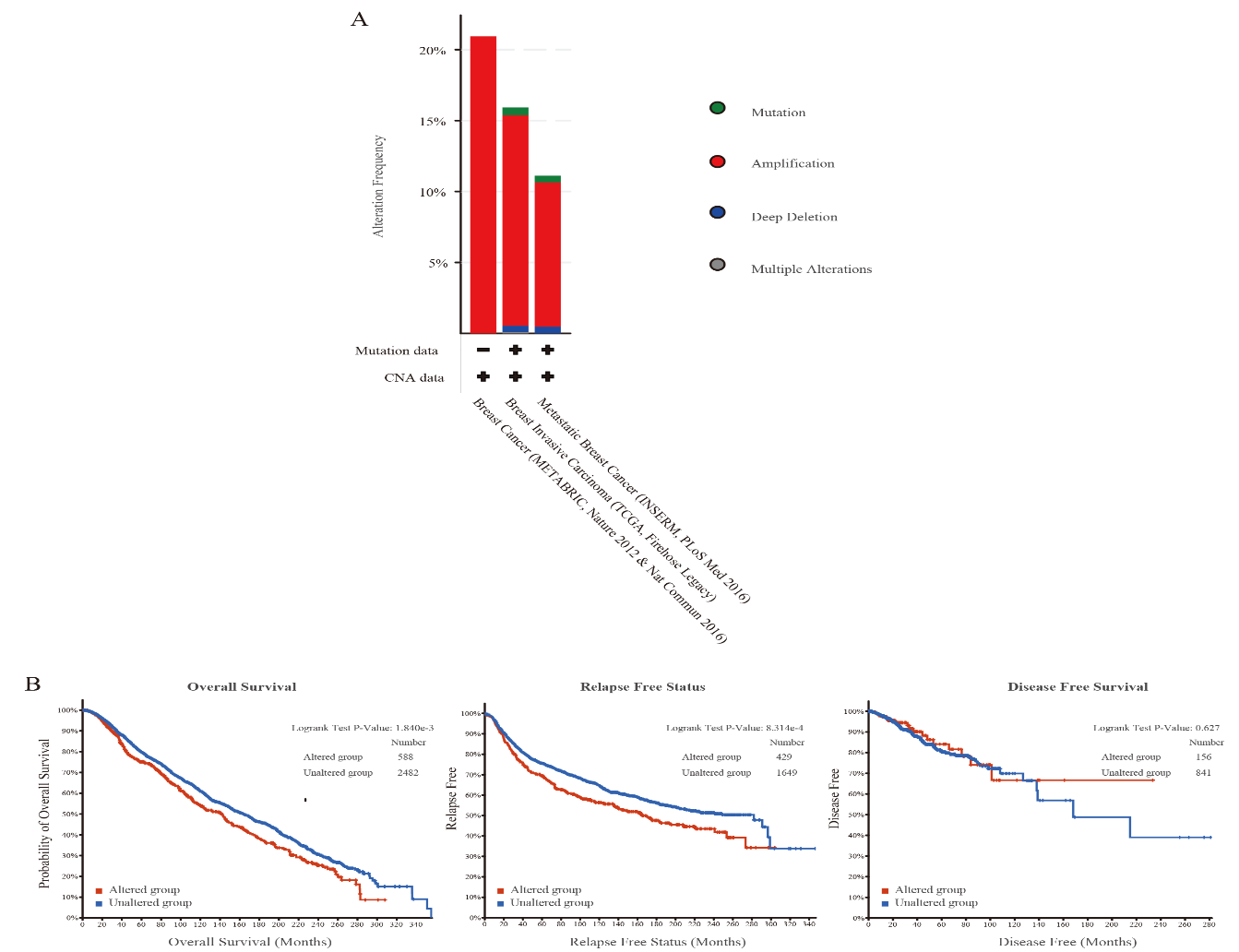


**Figure S3.** **GO analysis for PTK2.** The CC (A) and MF (B) of PTK2 were analyzed by LinkedOmics.


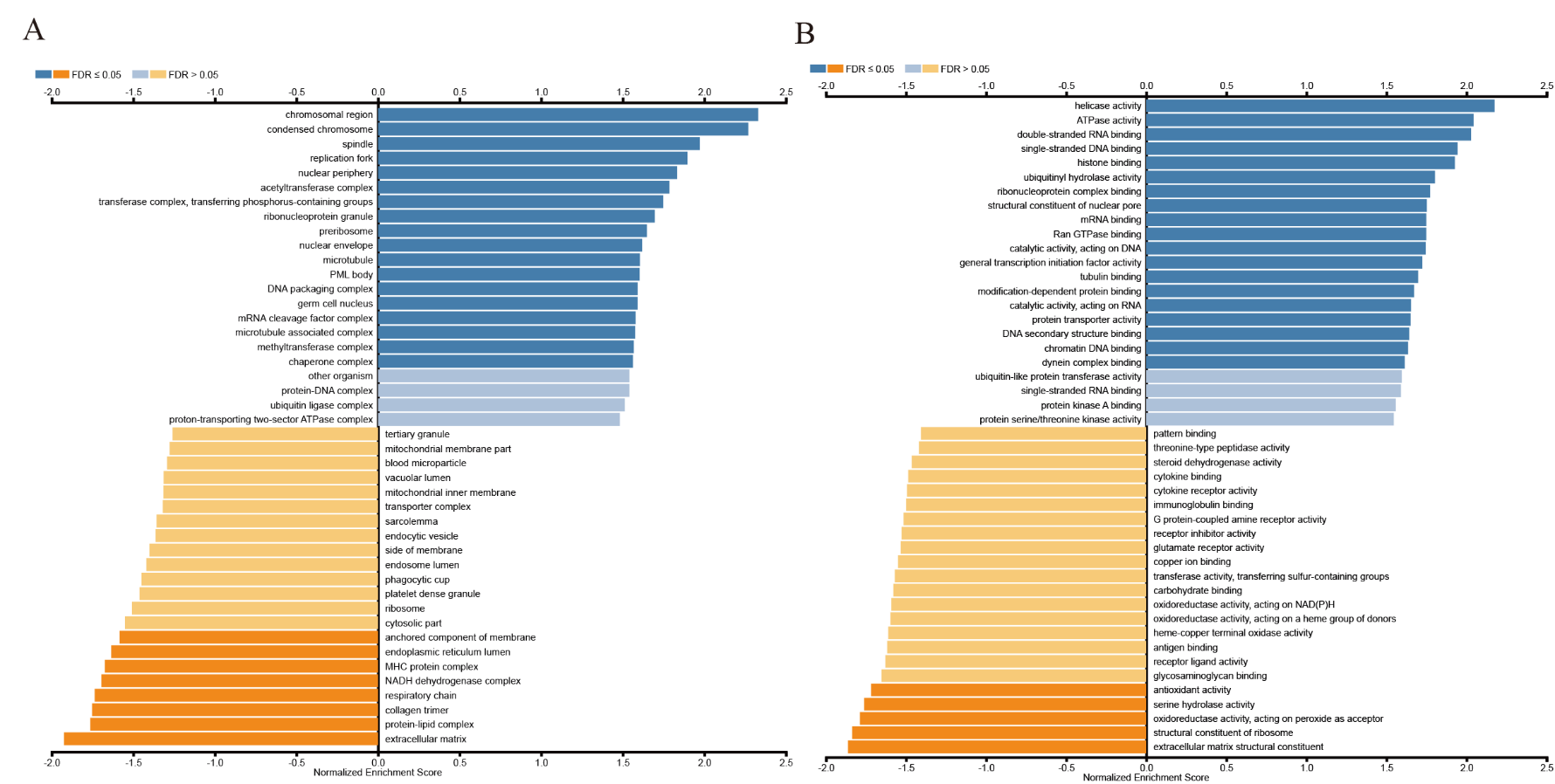

Supplement: Supplementary file 1 [file DataSheet1.docx]
